# A New Genus of Andean Katydid with Unusual Pronotal Structure for Enhancing Resonances

**DOI:** 10.3390/biology13121071

**Published:** 2024-12-20

**Authors:** Fabio A. Sarria-Sarria, Glenn K. Morris, Fernando Montealegre-Z

**Affiliations:** 1 School of Environmental and Life Sciences, University of Lincoln, Lincoln LN6 7DL, Lincolnshire, UK; fmontealegrez@lincoln.ac.uk; 2 Department of Biology, University of Toronto at Mississauga, Mississauga, ON L5L 1C6, Canada; glenn.morris@utoronto.ca

**Keywords:** morphological adaptation, biodiversity, sound waves, resonators

## Abstract

Katydids employ acoustic signals to communicate with others of their species and have evolved to generate sounds by coupling the anatomical structures of their forewings. However, some species have evolved to implement an additional resonance mechanism that enhances the transmission and sound pressure of the acoustic signals produced by the primary resonators. Secondary resonators, such as burrow cavities or horn-shaped structures, are found in the surrounding environment but could also occur as anatomical modifications of their bodies. Chamber-like structures have been described in species of katydids with modified pronota or wings. It has been shown that these modified structures directly affect the transmission and filtering of acoustic signals and can function as a Helmholtz resonator that encapsulates the primary sound source. By morphological and acoustic analysis, we describe a new genus of Conocephalinae and investigate the physical properties of their sound production structures for three new species from the Andes of Colombia and Ecuador. Males of the new genus, here described as *Tectucantus* n. gen., have a characteristic inflated pronotum enclosing the reduced first pair of wings and extending rearward over the first abdominal segments. We test the hypothesis that the pronotal cavity volume correlates with the carrier frequency of specific calls. The cavity of the pronotal chamber acts as a Helmholtz resonator in all three *Tectucantus* species and, potentially, in other distantly related species, which use similar secondary body resonators.

## Simple Summary

Katydid insects use sound to communicate through the frictional vibration of their wings. Secondary exoskeletal structures, evolved in proximity to a tooth row and scraper edge, include wing cells supported by veins (specula). These thin diaphragms are loaded by surrounding air spaces and may have evolved resonances contributing to signal power. In this study, three new species from the Andes are described and classified under a newly proposed genus. The males of the newly identified genus, *Tectucantus* n. gen., are mainly distinguished by their enlarged pronotum, which enhances the amplification of the produced sound.

## 1. Introduction

Acoustic signals are prevalent in the animal kingdom (mostly studied in vertebrates), and sound production behavior has evolved primarily to attract mates over long distances [1,2]. This behavior is often subject to the strong sexual and natural selection imposed by the environment, e.g., unintended receivers, such as predators and parasitoids [3,4]. As a result, animals have adapted a diverse range of morphological and behavioral strategies to ensure interspecific communication while avoiding eavesdropping species. Some of these adaptations have been achieved by taking advantage of the natural resonance of anatomical parts to increase the amplitude of acoustic signals [5]. Resonance occurs when the loading of an air mass (its acoustic impedance) is delimited by the structure, amplifying the vibrations of a system by matching its natural frequency [6]. Most such systems will have multiple natural resonances, with some frequencies amplified more than others [7]. Morphological adaptations include the mammalian larynx avian syrinx, [8] the “strigin” (see below) of insects and other structures resembling a Helmholtz resonator, such as burrow cavities [9,10] and body parts, including air sacs [11], modified wings or other chitinous structures [12,13,14].

Ensiferan insects (crickets, grigs, katydids) produce an airborne sound using their forewings through a frictional stridulation mechanism [15]. Their small wing-radiating areas present a challenge concerning the intended sound wavelength. To tackle this issue, they use resonance to alleviate the problem of size [4,16]. While the mechanics of a sound box or larynx do not apply in the case of crickets and allies, the resonance in Ensifera is exploited by stimulating the natural frequency of the wings using a file in one wing and a scraper in the other. The scraper is typically dragged across the file to strike a certain number of teeth per unit of time, matching the natural frequency of vibration of the wings [17,18]. For instance, if the wings are naturally tuned at 5 kHz, the wings are set in resonance, and the scraper strikes the file with a tooth rate of ca. 5000 teeth per second [19]. In addition to the first level of resonance, some Ensifera, like field crickets and grigs, use nearly symmetrical wings for sound radiation. Therefore, they maximize the area of sound radiation by using two synchronized wings [19] and an escapement mechanism [17,20]. However, most katydids have evolved asymmetrical wings for sound radiation, with the scraper wing being the primary wing for acoustic radiation [21].

Asymmetry has been interpreted as facilitating the production of pure-tone calls at much higher frequencies than those used by field crickets, including ultrasound [22]. However, this implies that katydids have lost one sound radiator and the area for efficient acoustic radiation. Katydids have, therefore, developed various adaptations to maximize the range of the high-frequency signal. One such adaptation is seen in their cuticular cavities or special wing designs that help produce louder signals [12,23]. An uncommon design involves pronotal chambers that cover all the wings or the relevant radiating wing cells [14]. Although there is no comprehensive evolutionary analysis of pronotal acoustic cavities, it is apparent that these structures evolved independently across the katydid family, as they appear randomly in species of different subfamilies [24,25].

This study introduces a new genus and three species of neotropical katydids found in the Andean cloud forests of Colombia and Ecuador. The males of this genus use different sound frequencies and have pronotal cavities that cover their reduced wings entirely. In addition to describing the new species, we also investigated the hypothesis that the size and volume of the pronotal chambers function as a neckless Helmholtz resonator and, therefore, are associated with the carrier frequency of the call in each species.

## 2. Materials and Methods

### 2.1. Specimen Collection

The collecting events occurred at night (18.00–22.00) along established trails in the sampling areas. Specimens were localized by sight or by following their calling song, mostly from trees and bushes along the forest paths. Specimens were captured by hand-herding into an insect net and then transferred individually to a 300 mL cylindrical plastic pot. Upon return to the base camp, the collected insects were placed in a cylindrical wire mesh cage and provided with pieces of fresh apple and water. The collected specimens were kept under the natural environmental conditions of the sampling area during the remaining period of the field expedition.

### 2.2. Localities

#### 2.2.1. Munchique NNP, Colombia

The Munchique National Natural Park is situated in the Cauca Department within the western mountain range of the Andean Region in Colombia. Located 61 km from Popayán in El Tambo, it shares boundaries with the Lopez, Morales, and Cajibio municipalities. The park covers an area of 440 km^2^, with elevations ranging from 200 m to 3500 m above sea level. The vegetation is mainly composed of trees that can exceed 40 m in height, but their numbers decline with elevation and epiphytes become more predominant. The average temperature is 24 °C at lower elevations and 8 °C at the highest points. The annual average rainfall is 3000 mm in the higher areas, increasing to 5000 mm in the lower regions [26].

#### 2.2.2. La Planada Natural Reserve, Colombia

The La Planada Natural Reserve is located in the Nariño Department (southwest Colombia), 27 km from the municipality of Ricaurte. It conserves approximately 3200 hectares of a montane rainforest. It is between 1300 and 2100 m above sea level. The average temperature is 20 °C. The annual average rainfall is 4700 mm, with a dry period between June and August [27].

#### 2.2.3. Santa Lucia Natural Reserve, Ecuador

The reserve is located in the Pichincha Province, 80 km northwest of Quito. It covers over 730 hectares of the montane cloud forest in the Chocó Andean Bioregion. It is between 1400 and 2500 m above sea level, and the environment consists of primary and secondary forests, reforested areas, and pastures [28]. The average temperature is 13 °C and it has an annual average rainfall of approximately 3000 mm [29].

#### 2.2.4. Bellavista Cloud Forest Reserve and Lodge

The Bellavista Lodge is situated in the Tandayapa Valley in the Pichincha Province, 52 km from Quito. It conserves approximately 374 hectares of a montane rainforest and is at 2000 m above sea level. The average temperature is 22 °C, and the annual average rainfall is 5000 mm [29].

### 2.3. Song Recordings

#### 2.3.1. Field Recordings

A total of 6 specimens were recorded in the field with audio-limited equipment, audio range (1–48 kHz). The field recordings in Munchique NNP (1997) and La Planada (2000) were obtained with a Sony Walkman WM D6C Professional cassette tape recorder with an ECM 909 microphone. The songs in the Santa Lucia Natural Reserve (2014) were recorded using a Tascam DR-05 Version 2 Dictaphone Linear PCM Portable Recorder (TEAC Corporation, Tokyo, Japan) and saved at a 96 kHz sampling rate. 

#### 2.3.2. Laboratory Recordings

Specimens (n = 13) were placed in cylindrical metallic mesh cages at a distance of 10 cm from the microphone (Figure S1). Full frequency range recording calls were acquired using a 1/8″ (Type 4138) or a 1/4″ (Type 4135) condenser microphone (Brüel & Kjær, Nærum, Denmark). The 1/8″ microphone was connected to a nexus amplifier (Brüel & Kjær, Nærum, Denmark) and to an acquisition software (PSV 10.0, Polytec GmbH, Waldbronn, Germany). A high pass filter was set at 1 kHz, with a sample frequency of 512 kHz. The output from the 1/4″ microphone was sent to a Racal instrumentation tape recorder running at 30“/s or was digitized (Tucker Davis, System II) at a sampling rate of 180 kHz and stored on a computer hard drive. Digitized signals were low-pass filtered at 90 kHz to avoid aliasing. A spectral analysis was performed on recorded songs using power spectral density estimates (discrete Fourier transform, nfft = 1024 points, Δf = 94 Hz) over individual syllables. The averages for the CF were calculated for each individual, and the resulting means were pooled to gain an overall mean and standard deviation, using Matlab (R2024a, The MathWorks, Inc., Natick, MA, USA).

### 2.4. Morphological Measurements

Body measurements were obtained with a Digital Vernier Caliper (Fowler, Newton, MA, USA). All measurements presented are in mm, following the measuring protocols used by Montealegre-Z and Morris [30]. Digital photographs of preserved stridulatory files were taken on an Alicona Infinite Focus microscope (Bruker Alicona, Graz, Austria). The measurements of inter-tooth spacing were obtained using CorelDraw X7 (Corel Corporation, Ottawa, ON, Canada) using the appropriate dimension tool; for details, see Montealegre-Z and Mason [21].

### 2.5. X-Ray Micro-Computer Tomography

X-ray Micro-Computer Tomography (µ-CT) scans of adult *Tectucantus* males were performed using a SkyScan 1172 µ-CT scanner (Bruker Corporation, Billerica, MA, USA) with a resolution of 9 µm (55 kV source voltage, 180 µA source current, 200 ms exposure and 0.2 deg rotation steps). Preserved specimens were placed upright in a small Eppendorf tube and positioned in a holder in the µ-CT scanner chamber. µ-CT projection images were reconstructed to produce stacks of orthogonal slices with NRecon (v. 1.6.9.18, Bruker Corporation, Billerica, MA, USA); the stacks were processed with CTAn (v. 1.15.4, Bruker Corporation) and virtual 3D models were built from the resulting images using Amira-Aviso 6.7 (Thermo Fisher Scientific, Waltham, MA, USA). For the full details of the method employed, see Jonsson et al. [13].

## 3. Results

### 3.1. Taxa Description

Order **ORTHOPTERA**

Family **TETTIGONIIDAE**

Subfamily **CONOCEPHALINAE** Kirby and Spence, 1826

Tribe **AGRAECIINI** Redtenbacher, 1891

#### 3.1.1. *Tectucantus* gen. nov. Sarria-Sarria, Morris and Montealegre-Z

Type species: *T. tinnulus* sp. nov. here described.

Etymology: This name is composed of the Latin terms *tectum* L., meaning roof, due to the characteristic pronotal formation covering the forewings, and *cantus* L., meaning song, which refers to “sheltered-song”.

**Figure 1:**
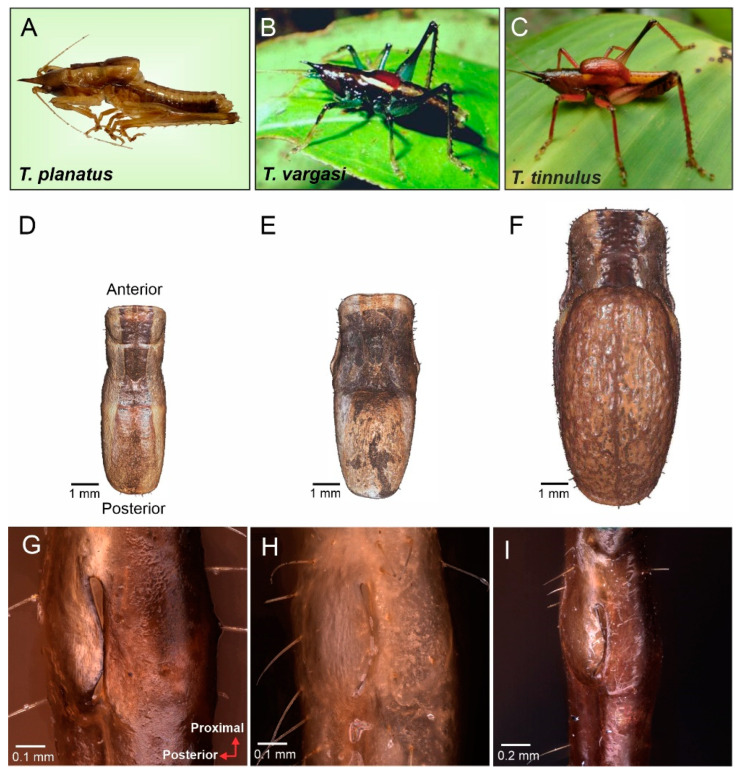
Morphological characters of *Tectucantus* spp. (**A**–**C**) Habitus of male of *T. planatus*, *T. vargasi*, and *T. tinnulus*. (**D**–**F**) Dorsal view and size comparison of pronotum. (**G**–**I**) *Tectucantus* spp. tympanal slits design.

**Figure 2:**
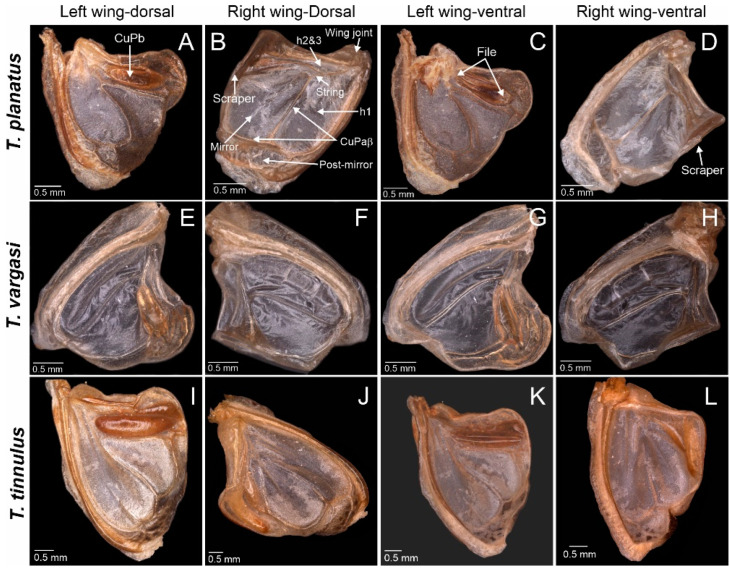
*Tectucantus* spp. wing morphology, dorsal and ventral view. (**A**–**D**) *T. planatus* (**E**–**H**) *T. vargasi* (**I**–**L**) *T. tinnulus*.

Diagnosis: This new genus is distinguished from the other Agraeciini by the following combination of morphological characteristics: The male pronotum possesses enlarged mesozona and metazona covering the forewings (Figure 1D–F). Forewings reduced, hind wings absent. The mirror and h1 cell area large and proportional, wholly occupying the wing’s effective area for sound radiation (Figure 2). The foretibiae show anterior and posterior tympana covered by cuticular pinna. The tympanal slit not entirely open; tympanal flaps fused in the middle to the dorsal cuticle, and at each end. Tympanal slits shape resemble a small drop (Figure 1F,G).

**Figure 3:**
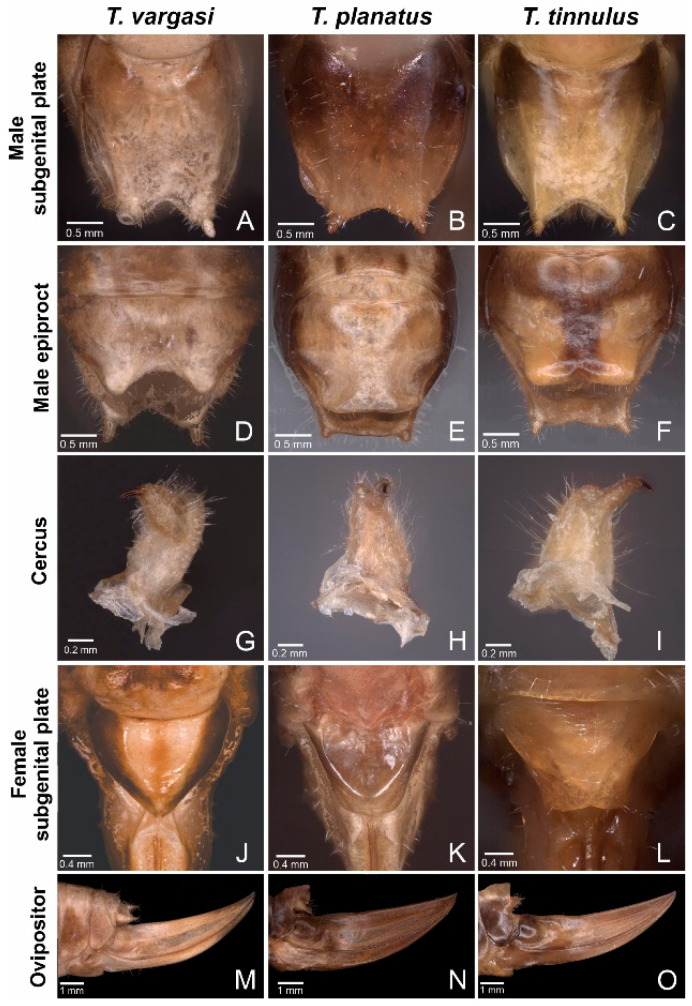
*Tectucantus* spp. abdominal morphological features. (**A**–**C**) Ventral view of male subgenital plate. (**D**–**F**) Dorsal view of male epiproct. (**G**–**I**) Male right cercus. (**J**–**L**) Dorsal view of female subgenital plate. (**M**–**O**) Side view of female ovipositor.

Description: Light brown to brown coloration, with sufflavus bands and dark brown markings and patterns. Head prognathous, conical (Figure 1). Fastigium length is half a distance between vertex and apical part of labrum. Basal portion of fastigium as broad as the scapus, with a sharp apex slightly down-curved (Figure 1A–C). Eyes prominent and globose. Scapus longer 2× wider than pedicel. Frons flat and smooth, lacking cuticular features. Frontal ocellus inconspicuous. Mandibles and clypeus symmetrical. Ovipositor as long as the head and pronotum combined, up-curved and moderately broad, with an acuminate tip (Figure 3M–O).

Thorax: Dorsal surface of pronotum smooth, approximately 2.5 times longer than wide, extending over the mesothorax and metathorax. Prozona saddle-shaped, does not cover propleura, leaving the acoustic spiracle visible. Mesozona and metazona dome-shaped, covering forewings (Figure 1A–C). Thoracic auditory spiracle small and circular.

Legs: Anterior and posterior genicular lobes of all femora armed with a spine. Hind femora much more prominent at the base, abruptly tapering distally. Tympanal slits curved posteriorad, with the anterior and distal ends opened, and middle region constricted by dorsal and lateral flaps in total or partial contact (Figure 1E–G).

**Figure 4:**
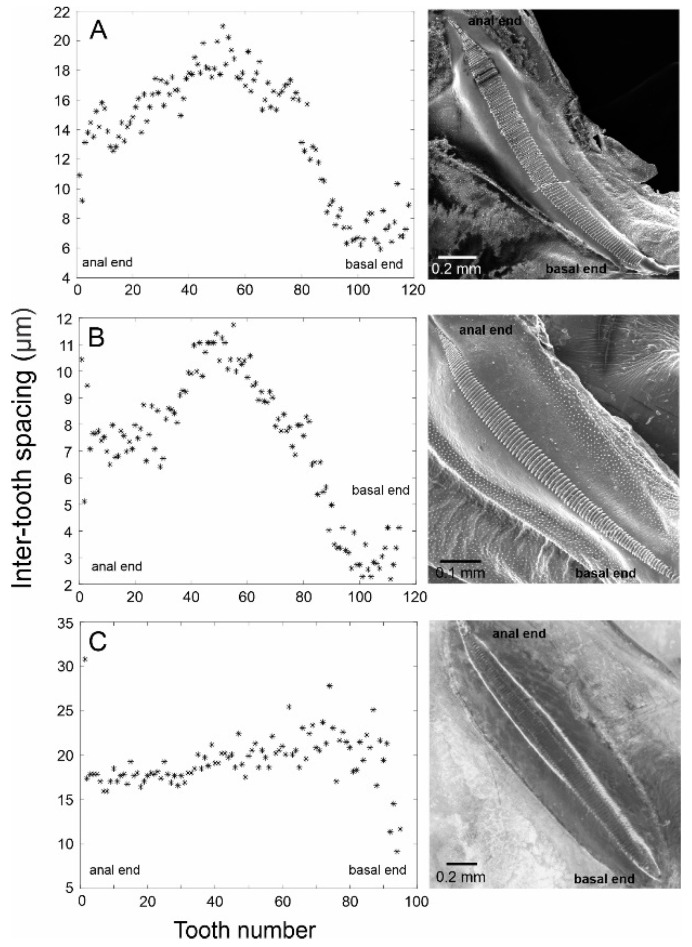
The stridulatory file of *Tectucantus* spp. Graph panels on the left show the measurements of inter-tooth distances in the direction of scraper motion during stridulation (anal to basal), and the panels on the right show SEM pictures of the files of each species, except for *T. tinnulus*. (**A**) *T. planatus*, (**B**) *T. vargasi*, and (**C**) *T. tinnulus*.

Wings: Males brachypterous, wings reduced to the function of sound production, and modified wing cells and amplification structures: mirror cell, which is often thoroughly transparent, and h1 cell (Figure 2). Male tegmina half the length of the pronotum. Hindwings absent. Females apterous. Stridulatory file narrow and straight in the anterior half, curving laterally and tapering to a point in the posterior half (Figure 4; teeth similar in thickness but become narrow in the posterior portion; tooth count ranges from 96 to 120.

#### 3.1.2. *Tectucantus tinnulus* sp. nov. Sarria-Sarria, Morris and Montealegre-Z

Etymology: Tinnulus is from the Latin term for ‘ringing’ or ‘tinkling’, a reference to the tonal quality of the call as affected by the resonant chamber.

Diagnosis: The species is recognized by the large male pronotal chamber, cerci shape, stridulatory file tooth arrangement and call peak carrier frequency.

**Figure 5:**
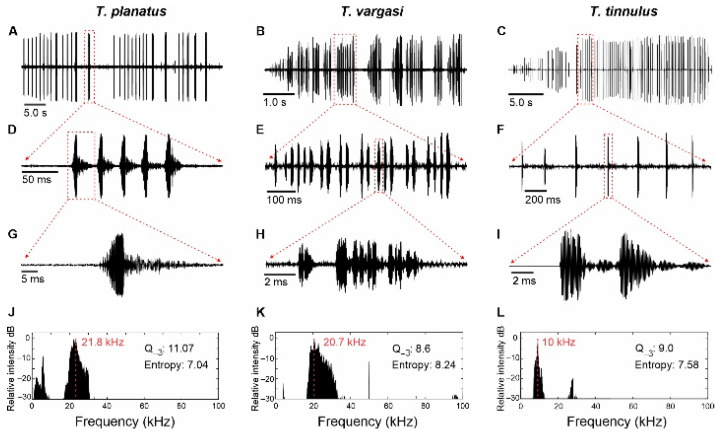
Acoustic analysis of *Tectucantus* spp. (**A**–**C**) Calling song of a recorded section. (**D**–**F**) Close-up view of an echeme. (**G**–**I**) Close-up view of a syllable (**J**–**L**) Frequency spectrum of a single syllable, red dashed line indicates peak frequency.

Description: Pronotum—length ca. 10.8 mm and pronotal cavity ca. 63.95 mm^3^. Wings—Right mirror and h1 cell area ca. 5.04 mm^2^ (Figure 2J,L). The stridulatory file bears 95–100 teeth. Inter-tooth spacing varies, as shown in Figure 5. Abdomen—Male tenth tergite superficially bilobate, forming a V-shaped notch (Figure 3F). Male cerci basally broad, incurved, with distal half elongated, and tip acuminated and strongly sclerotised (Figure 3I). Male subgenital plate quadrangular with a V-shaped notch, bearing two minuscule styli, hardly differentiated from the distal plate contour (Figure 3C). Female subgenital plate subtriangular, distally rounded, with a minute medial notch (Figure 3L). See Table 1 for the anatomical measurements.

Head, eye, and base of the fastigium fuscus, distal half of the fastigium with a furvus brown color (Figure 1C). Dorsal surface of the pronotum prozona bears a fulvous brown band, lateral lobe rubiginosus brown. Pronotal dorsal areas of the mesozona and metazona distinctively rubiginosus brown with two narrow fulvous brown stripes on the side, dividing the aurantius brown color of the lateral surface. Femora rubiginosus brown, turning into a furvus brown at the apex. Scape and pedicel furvus brown, antennae filaments exhibit spadix brown coloration. Abdomen tergites aurantius brown with a row of small dark brown spots on each side of the abdomen, the rest of the abdomen fuscus brown.

Material examined: Holotype: 1♂, Ecuador, Pichincha, Nanegal, Reserva Santa Lucía. September 2014 (F. A. Sarria-S and F. Montealegre-Z) MEUV. Allotype: 1♀, Ecuador, Pichincha, Nanegal, Reserva Santa Lucía. September 2014 (F. A. Sarria-S and F. Montealegre-Z) MEUV. Paratypes: 2♂♂, Ecuador, Pichincha, Nanegal, Reserva Santa Lucía. September 2014 (F. A. Sarria-S and F. Montealegre-Z) Bioacoustics and sensory Biology lab insect collection, University of Lincoln.

#### 3.1.3. *Tectucantus vargasi* sp. nov. Sarria-Sarria, Morris and Montealegre-Z

Etymology: Vargasi, in recognition of our colleague Fernando Vargas-Salinas, who assisted in numerous field expeditions in the Colombian Andes.

Diagnosis: This species is recognized by the male coloration pattern, genitalia, stridulatory file tooth arrangement and call peak carrier frequency.

Description: Pronotum—length ca. 7.8 mm, pronotal cavity ca. 15.22 mm^3^. Wings—right mirror and h1 cell area ca. 2.17 mm^2^ (Figure 3F,H). Stridulatory file ca. 0.8 mm in length, bearing 110–115 teeth. Inter-tooth spacing varies, as shown in Figure 5. Abdomen—male subgenital plate quadrangular, bearing two small styli and differentiated from the distal plate contour (Figure 4A). Tenth tergite bilobate, forming a U-shaped notch (Figure 3D). Male cerci basally broad, in-curved, with their distal half elongated, and tip acuminated and strongly sclerotised (Figure 3G). Female subgenital plate subtriangular, distally acuminated (Figure 3J). See Table 1 for the anatomical measurements.

Head, eyes, and fastigium furvus brown, prozona of pronotum fuscus brown, dorsal area of mesozona and metazona rubidus red with two sufflavus lateral stripes (Figure 1B). Femora varies in coloration, from thalassinus blue to fuscus brown at the apex. Tibia proximal end fuscus brown, distal end aureus. Scape and pedicel thalassinus blue, antennae filaments vitellinus. Abdomen fuscus brown with two spadix brown dorsal stripes.

Material examined: Holotype: 1♂, Colombia, Cauca, Parque Nacional Natural Munchique. 23 May 2000 (F. Montealegre-Z, F. Vargas-S, and L. Castaño) MEUV. Allotype: 1♀, Colombia, Cauca, Parque Nacional Natural Munchique. 23 May 2000 (F. Montealegre-Z, F. Vargas-S, and L. Castaño) MEUV. Paratype: 1♂, Colombia, Cauca, Parque Nacional Natural Munchique. 23 May 2000 (F. Montealegre-Z, F. Vargas-S, and L. Castaño) Bioacoustics and sensory Biology lab insect collection, University of Lincoln.

#### 3.1.4. *Tectucantus planatus* sp. nov. Sarria-Sarria, Morris and Montealegre-Z

Etymology: Planatus, refers to the locality where the specimens were collected and also relates to the shape of the male pronotum, which is the least inflated of the three species described for the genus.

Diagnosis: The species is recognized by the male coloration pattern, pronotal chamber size, genitalia, stridulatory file tooth arrangement, male epiproct and call peak carrier frequency.

Description: Pronotum—length ca. 7.5 mm and pronotal cavity ca. 13.2 mm^3^. Wings—right mirror and h1 cell area ca. 2.23 mm^2^ (Figure 2B,D). Stridulatory file ca. 1.6 mm in length, bearing 115–120 teeth. Inter-tooth spacing varies, as shown in Figure 4. Abdomen—Male subgenital plate quadrangular, bearing two small styli, differentiated from the distal plate contour (Figure 3B). Tenth tergite truncated, forming a straight distal margin (Figure 3E). Male cerci basally broad, in-curved, distal half s elongated, with a small process dorsally, tip acuminated and strongly sclerotised (Figure 3H). Female subgenital plate subtriangular and distally truncated (Figure 3K). See Table 1 for the anatomical measurements.

Coloration: In preserved specimens, head, eyes, and base of the fastigium brunneus brown. Dorsal surface of pronotum with a fuscus brown band on the prozona, extending and widening over the mesozona and metazona, lateral lobe area of the pronotum bearing an armeniacus brown band (Figure 1). Femora and tibiae armeniacus brown. Scape, pedicel, and antennae aurantius brown. Abdominal tergites armeniacus brown, with a central brunneus brown band, and fuscus brown margins, Abdominal pleura fuscus brown.

Material examined: Holotype: 1♂, Colombia, Nariño, Reserva Natural La Planada. 3 May 1997 (F. Montealegre-Z) MEUV. Allotype: 1♀, Colombia, Nariño, Reserva Natural La Planada. 2 April 1997 (F. Montealegre-Z) MEUV. Paratype: 1♂, Colombia, Nariño, Reserva Natural La Planada. 30 May–10 June 2001 (F. Montealegre-Z, F. Gomez) Bioacoustics and sensory Biology lab insect collection, University of Lincoln.

### 3.2. Bioacoustics

**Figure 6:**
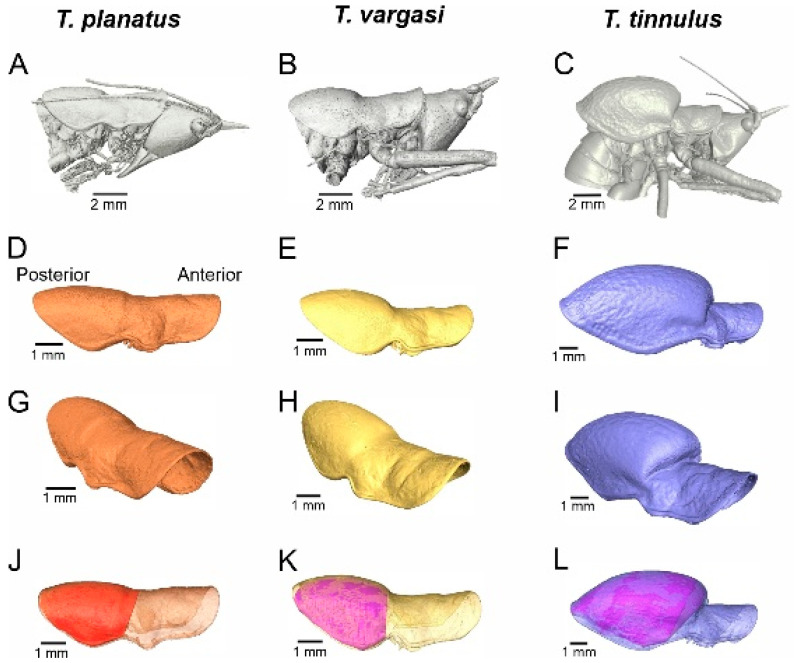
Three-dimensional segmentation and volumetric features. (**A**–**C**) Side view of head and thorax. (**D**–**L**) Pronotum 3D reconstruction, side view and pronotal cavity volume. (**J**–**L**) Pronotal volume represented by the shaded area.

The males of *Tectucantus* spp. stridulate continuously and their calls last between 6 and 40 s, depending on the species. The song structure consists of a series of echeme sequences or syllable trains (Figure 5), and individual syllables contain 7–10 sets of sustained sinusoidal pulses. The Q value (3 dB down of the dominant peak) is in the range of 8.6 to 11.07 and the spectral entropy (signal complexity: tonal vs. broadband) is in the range of 7.0 to 7.58. *Tectucantus* species have a notable tendency for producing pure tone calls, particularly observed in *T. tinnulus*. In this species, the file exhibits a relatively flat shape with a gradually increasing and dense arrangement of teeth, contributing to its tonal quality [31]. Conversely, the file takes on a more convex curvature at its midpoint in *T. vargasi* and *T. planatus* (Figure 4A,B). This curvature alters the distribution and arrangement of the teeth along the file and affects sound quality, producing calls with a broader spectrum (Figure 5); this effect has been discussed in Montealegre-Z and Mason [21] for several Panacanthus species (see figure 6 of that article).

#### 3.2.1. *Tectucantus tinnulus* sp. nov.

The call of this species has been described by Morris and Mason [14] as “a repetition of zips (noisy sounds < 1 s long), perceived by the human listener as having infrastructure”. The zips are interpreted here as syllables in the specimens collected in Santa. Lucia Ecuador; these syllables have a duration of 15–26 ms (17 ± 12 ms) and an average of 5.6 ± 2.5 discrete pulses (range 2–13). According to the values reported by Morris and Mason [14], the syllables had a duration of 35.7 ± 7.4 ms and an average of nine discrete pulses (range 7–12). A common feature across all of the specimens recorded is that the call incorporates groups of two syllables given quickly, as observed in lab and field recordings.

For individuals from Santa Lucia, the song has a peak frequency of about 10.21 kHz (Figure 5L). This frequency is about 1.5 kHz higher than that reported by Morris and Mason [14] for specimens collected near the volcano in Pichincha (~8.85 kHz).

All of the specimens measured and recorded in different decades show a spectral energy within a relatively narrow frequency band between ca. and spectral breadth ranging, from 7 to 13 kHz at −30 dB below its peak. This band is narrower than the one obtained for the other species (see below). The recordings under lab conditions exhibit an additional low-amplitude frequency peak, at 26–29 kHz, that is not present in field recordings. Morris and Mason [14] and Jonsson et al. [13] reported similar ultrasonic frequency components, assuming that the higher frequencies are present in all songs but attenuate quickly under field conditions. The function of these ultrasonic components in these species is unknown, but in other species of katydids, high energy peaks seem to provide a means for males to judge the distance of other singing males [32,33]. In field crickets, high frequency harmonics seem to enhance directional hearing [34,35].

#### 3.2.2. *Tectucantus vargasi* sp. nov.

The calling events consist of groups of echeme sequences, each containing 17 ± 9 syllables (range 9–38) (Figure 5B,E). Each echeme lasts for approximately 587 ± 383 ms and the syllable duration varies between 4 and 15 ms (9.6 ± 2 ms), consisting of a sequence of 2–16 discrete pulses (10.2 ± 3.6 pulses). The echeme sequence period ranges between 112 and 454 ms (237 ± 136 ms), but this is highly variable within the same individual and a period reduction down to ~100 ms is usually observed at the start of the echeme sequence. The song has a peak frequency of 20.7–21.2 kHz (Figure 5K) and the spectral breadth ranged asymmetrically from ~17 to 38 kHz at −30 dB below its peak.

#### 3.2.3. *Tectucantus planatus* sp. nov.

The calling events consist of groups of echeme sequences, each containing 5.3 ± 0.5 syllables (range 5–6, Figure 5A,D). Each echeme lasts for approximately 175 ± 18 ms (Figure 5D). The syllable duration varies between 2 and 16 ms (7.6 ± 2.6 ms) and consists of a sequence of discrete pulses in the first 6 ms, followed by a sustained oscillation of about 3–4 ms (Figure 5G). The echeme period varies between 231 and 965 ms (817 ± 230 ms), although, occasionally, the same individual increases the rate of the echeme, reducing the period to about 200 ms. The song has a peak frequency of 21–23 kHz (Figure 5J), and the spectral breadth ranges asymmetrically from 19 to 30 kHz at −30 dB below its peak.

### 3.3. Estimation of Resonant Frequency Based on 3D Pronotal Cavity Volume

Figure 6 shows the segmented 3D models of the pronotal cavities reconstructed using the µ-CT scan data. Through these segmentations, it was possible to quantify the volume and dimensions of the opening area of the cavity for each species (Table 2). Nevertheless, the volume estimated for the pronotal area should be considered a maximum approximation since the thorax and wings occupy a portion of the volume.

The resonance frequency of the structure can be calculated by incorporating parameters such as the speed of sound (*C*, 343 m s^−1^), the opening area (*A*), the cavity volume (*V*) and the length of the neck (*l*) into the Helmholtz resonator equation [13,14].

(1) $$f_{h}=\frac{C}{2\pi }\sqrt{\frac{A}{Vl}}$$

In specific configurations, such as the pronotal modification observed in *Tectucantus* spp., the neck is absent, and an end-correction term is used to modify the equation to calculate the resonance frequency, f_h_, of a typical Helmholtz resonator [36].

(2) $$f_{h}=\frac{C}{2\pi }\sqrt{\frac{1.85r}{V}}$$

**Figure 7:**
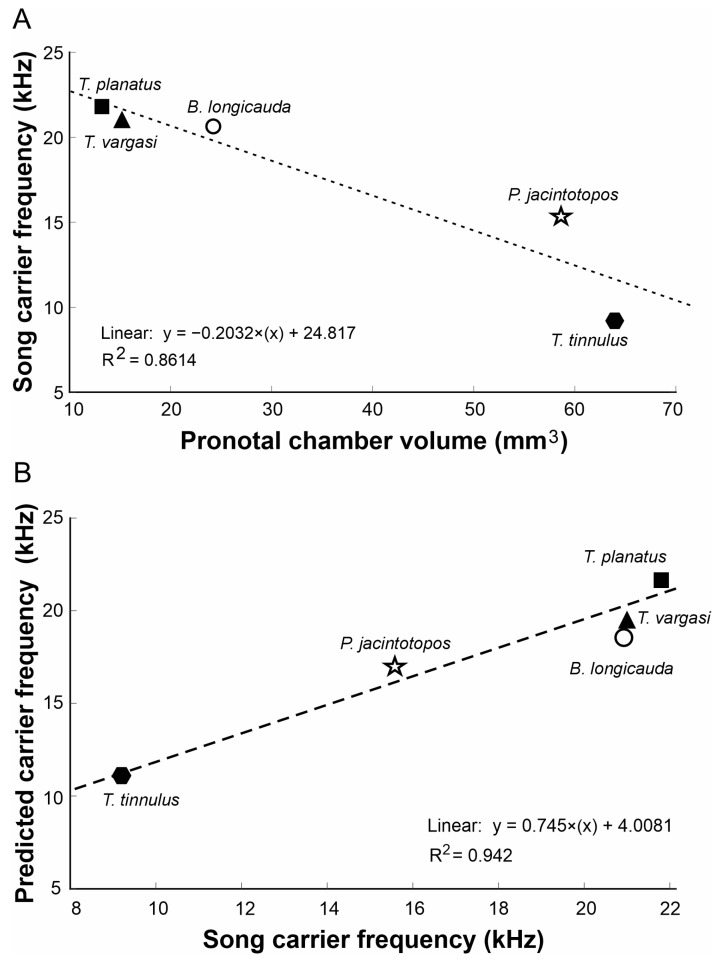
Relationships between *Tectucantus* spp. pronotal cavity and calling songs. (**A**) Correlation between pronotal volume and carrier frequency. (**B**) Correlation between carrier frequency and predicted carrier frequency.

**Figure 8:**
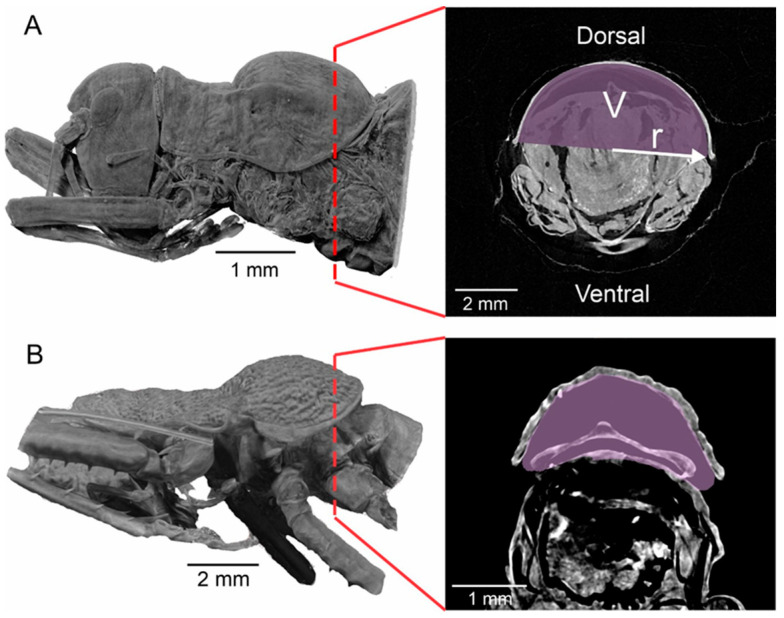
Three-dimensional segmentation and orthoslice features. (**A**) *P. jacintotopos* thorax side view and orthoslice. (**B**) *B. longicauda* thorax side view and orthoslice. V: volume, r: radius.

The neckless Helmholtz resonator Equation (1) was applied to examine the relationship between the carrier frequency of male calling songs and the volume and geometric dimensions of the pronotal cavity. Table 2 presents comprehensive data for the three species under investigation, including specific measurements of pronotal cavity volume, opening radius, the observed carrier frequency of the calling song, and the estimated frequency derived from the resonator equation. This detailed analysis aims to identify any correlations between the physical characteristics of the pronotal cavity and the frequencies emitted in the males’ calling songs. Figure 7 illustrates a strong correlation between pronotal volume and song carrier frequency. As a proof of concept, we included additional species with inflated pronota (Figure 8), such as *Phymonotus jacintotopos*, an endemic species from California [23] and *Bispinolakis longicauda*, a species from the eastern Malaysia highlands [37]. Using the pronotal chamber volume of these species, we investigated a theoretical frequency value based on the regression equation shown in Figure 7A. The correlation was significant (Df = 1; Sum sq.= 98.171; Mean sq.= 98.171; F = 18.643 (1,1); *p*-value= 0.0228). These species have a song carrier of 15.6 kHz and 20.8 kHz, respectively, but the predicted carrier frequencies, using the equation in Figure 7A, indicate a value of 10.5 and 18.9 kHz, nearly 5 and 1 kHz lower. However, we regressed the calculated frequency based on the neckless Helmholtz resonator equation in the insect carrier to validate the method, assuming that the correlation will be very high (Figure 7B). This correlation was also significant (Df = 1; Sum sq.= 63.258; Mean sq.= 63.258; F = 49.035 (1,1); *p*-value= 0.006). Using this equation, we predict a pronotal cavity resonance of 15.6 kHz for *P. jacintotopos*, matching the reported carrier frequency for this species [23], and 19.8 kHz for that of *B. longicauda*.

## 4. Discussion

### 4.1. Taxonomy

The morphological comparison shows *T. vargasi* and *T. planatus* sharing more morphological features than either of them do with *T. tinnulus*. This includes not just the pronotal shape but also the design of the stridulatory file (Figure 4) and genital characters, like the male cerci and female subgenital plate (Figure 3). This suggests that *T. tinnulus* exhibits more derived characteristics. Still, this question requires a deeper phylogenetic analysis in light of molecular techniques. The above also implies that *T. tinnulus* have evolved sound features from their ultrasonic ancestors, and the reduction in carrier frequency might have resulted, in part, from the increase in body size, which affects wing sizes and wing resonances, and the volume of the air space in the pronotal cavity. Evolving to have low frequencies from high-frequency ancestors have been shown in other katydids, such as *Panacanthus* spp. for example [38].

To which tribe should *Tectucantus* be assigned? Like *Tectucantus* spp., Cestrophorini is a tribe of small agraeciine-like katydids found in Ecuador’s montane rainforests. They are known for their distinctive fastigium and exposed tympanic membrane on the foretibiae [39]. This group includes two genera: *Cestrophorus* Redtenbacher (1891) and *Acanthacara* Scudder (1869). *Tectucantus* differs from these genera in them having covered tympana and modified pronotum in males, which conceals the reduced tegmina. Furthermore, *Tectucantus* spp. closely resembles *Bispinolakis* Ingrisch, 1998 (Agraeciini: Liarina), an Asian monotypic genus incorporating *Bispinolakis longicauda* [37]. They are similar in having inflated pronotum, narrow fastigia, almost symmetrical tegminae, and call structure given by syllable trains. It remains unclear whether these similarities arise from homology or convergent evolution; thus, a robust molecular phylogeny is necessary for clarification. Consequently, we propose classifying Tectucantus within the Agraeciini, based on its acoustic and morphological features, until further molecular analyses can be conducted.

### 4.2. Bioacoustics and Pronotal Cavity as a Secondary Resonator

The behavior during stridulation could significantly contribute to sound production by adjusting the posture (tilt) of the pronotum and, thus, the pronotal cavity opening. We do not have enough information about how males of this group could adjust the back opening of the pronotum during singing, but this is a possibility, mostly under ambient temperature changes. However, any adjustment should occur within the tolerance range allowed by wing resonances. This system demonstrates comparable features to the vocal mechanisms present in vertebrates, such as the larynx and syrinx. In these anatomical structures, the vocal tract culminates in a flexible cavity—either the mouth or the beak—that can be adjusted to generate emphasized formants in the emitted sound [40]. Notably, the katydid’s distinctive “stringing” mechanism stands out due to its exceptional efficiency, producing a sound output of 85 dB at a distance of 10 cm [14]. This achievement is particularly significant given the small size of these insects. Moreover, this resonant behavior appears largely independent of the physical properties of the chamber itself. As Jonsson et al. [13] demonstrated, the pronotal chamber behaves as a Helmholtz resonator, reinforcing the frequencies produced by the forewings. The resonant frequency of a Helmholtz resonator is determined by its volume, length and area of the neck. This was also shown in the auditory pinnae cavity of other tettigonids [41]. This suggests that the effective functioning of the chamber relies more on its geometric configuration and the dynamics of the air within it, rather than on the materials from which it is constructed.

To demonstrate our approach, we explored predicting the sound carrier frequencies of *P. jacintotopos* and *B. longicauda* using a regression analysis based on the data from the three species of *Tectucantus* (see Figure 7). However, the predicted carrier frequencies of 10.5 kHz and 19.5 kHz were approximately 5 kHz and 1 kHz lower than the fundamental frequencies of these species, which are 15.6 kHz and 20.8 kHz, respectively. This discrepancy could result from using only three data points to obtain the regression, which could cause predictions to be biased and not include more interspecific variability, or, in the case of *P.*, its *jancitotopos* pronotal shape differing from those of *Tectucantus* spp. and *B. longicauda* in having a larger and more exposed opening. Nevertheless, predicting the pronotal cavity resonance on carrier frequency could produce a more accurate prediction (as suggested by R^2^ (Figure 7B)) when extrapolating the carrier frequency of *P. jacintotopos*. This emphasizes the need for an in-depth study of secondary couple resonators in body cavities using appropriate phylogenetic regressions and incorporating the methods described here.

### 4.3. Optimization for Resonance as an Adaptation Driven by Environmental Stress

Most tettigoniid forewings function (at different times) in both the locomotor act of flying and the communicative act of resonance filtering, i.e., they have evolved body structures to optimize for both effective flight and resonant stridulation. But many katydids spp., like *Tectucantus*, no longer use their wings for flight, so they are free of the selective environmental stresses that serve to optimize flying locomotion. Their wings only need to respond to environmental stress that favors resonance.

The primary environmental factor influencing the adaptation of resonance is the necessity of being heard by mates or rivals in a noisy environment where other animals, especially conspecifics, communicate using a similar frequency range [42]. In tropical environments, the diversity in signal carriers is high, and background noise can exceed 70 dB [43]. Consequently, the development of resonators for amplifying acoustic signals could have been favored by natural selection [44]. A subtle boost in amplitude, of just a few decibels, can significantly diminish the effect of background noise on the intended signal. This adjustment enhances the acoustic signal and extends its effective operational range, allowing for better communication in noisy environments [45]. It is an act of filtering out noise.

The natural resonance of cuticular structures and first resonators, unimpeded with any role in flight, will be tightly affected by the thickness, shape, and elasticity of their constituent (composite) material (chitin) [46]. Where the exoskeleton enhances the leverage of flying locomotion, there will be a premium on adaptive elasticity (stiffness) at different skeletal loci, one favoring the effectiveness of the cuticle at that locus in translating and conducting forces. Thus, flightless species of katydids, without second resonators, have adapted modifications on their reduced wings for enhancing sound production at high frequencies [47,48]. Likewise, *Tectucantus* species, as well as *P. jacintotopos* and *B. longicauda*, being flightless, are more unrestricted in allowing stress (in the form of environmental masking noise) to optimize a natural resonant frequency at a non-overlapping carrier.

## 5. Conclusions

Our findings support the hypothesis that the pronotal chamber functions as a Helmholtz resonator in all three identified *Tectucantus* species. Additionally, it is significant that this resonating function may also be present in other, albeit distantly related, species that share similar structural features in their pronotum. This underscores the need for a comprehensive investigation into secondary coupling resonators associated with body cavities and, together with the appropriate phylogenetic regression techniques, to attain a thorough understanding of these resonators and their implications in acoustic communication as a response to environmental stress. The approach presented here could be applied in future research involving museum specimens with similar structures. It is non-invasive and enables the estimation of acoustic components related to morphological adaptations, improving our understanding of evolutionary processes over a wider taxonomic and temporal range.

## Figures and Tables

**Table 1:** Body measurements. All measurements are in mm. For paired features (e.g., legs), left/right features were reported, respectively. n.a. = not applicable, where the structure was not present to be measured (e.g., damaged, absent, or absent due to sex differences).

Species	*T. planatus*				*T. vargasi*				*T. tinnulus*			
Body Part	♂ (*n* = 5)	SD	♀ (*n* = 2)	SD	♂ (*n* = 3)	SD	♀ (*n* = 1)	SD	♂ (*n* = 6)	SD	♀ (*n* = 1)	SD
Fastigium	2.18	0.3	2.4	0	2.23	0.28	2.5	0	2.92	0.13	2.63	0
Fastigium base	0.82	0.07	0.81	0.08	0.7	0.07	0.88	0	0.92	0.05	0.95	0
Frons	3.41	0.05	3.88	0.18	3.25	0.28	3.7	0	3.89	0.12	4.3	0
F-Femur	4.98	0.11	5.56	0.05	4.82	0.02	5.25	0	5.65	0.07	5.8	0.07
F-Tibia	5.16	0.11	5.78	0.11	5.07	0.25	5.3	0.07	6.11	0.06	6	0.35
M-Femur	4.95	0.14	5.44	0.48	4.69	0.16	4.95	0.07	5.31	0.11	5.45	0.07
M-Tibia	5.26	0.01	5.6	0.11	4.9	0	5.38	0.18	6	0.11	6.25	0
H-Femur	9.03	1.51	10.6	0.28	9.07	0.17	10.15	0.21	10.03	0.11	10.55	0.07
H-Tibia	9.16	1.39	11.2	0.07	9.18	0.11	10.1	0.14	10.18	0.06	11.15	0.21
Eye	0.9	0.02	0.94	0.01	0.8	0.04	0.9	0	0.9	0.01	0.99	0.02
inter eye space	2.03	0.07	2.25	0	2.08	0.16	2.1	0	2.32	0.1	2.38	0
Pronotum length	6.56	0.2	4.68	0.25	6.55	0.18	4.5	0	9.23	0.4	4.3	0
Pronotum width	3.08	0.14	3.63	0.53	3.13	0.38	3.25	0	3.63	0.13	3.95	0
Subgenital plate length	2.16	0.28	1.2	0	2.16	0.4	1.25	0	2.24	0.1	1.5	0
Subgenital plate width	1.96	0.16	1.5	0.35	1.87	0.4	1.62	0	2.24	0.07	2	0
Epiproct	1.47	0.07	0	0	1.08	0.19	0.62	0	1.24	0.06	0.5	0
Abdomen length	9.28	1.23	12.25	1.48	7.83	1.53	7.5	0	8.57	1.13	11	0
Ovipositor	n.a.	n.a.	7.1	0.57	n.a.	n.a.	6	0	n.a.	n.a.	8	0
Ovipositor base	n.a.	n.a.	2	0	n.a.	n.a.	1.75	0	n.a.	n.a.	2.25	0

**Table 2:** Estimated carrier frequency using Equation (2) for a neckless Helmholtz resonator.

Species	Volume (mm^3^)	Radius (mm)	CF Song (kHz)	Predicted Resonant Frequency (kHz)
*T. tinnulus*	63.95	1.39	9.5	10.4
*T. vargasi*	15.22	1.05	21	19.5
*T. planatus*	13.2	1.06	21.8	21
*B. longicauda*	24.48	1.47	20.8	18.2
*P. jacintotopos*	58.71	3.02	15.6	16.8

## Data Availability

The 3D models of pronotum chambers are available as STL files, and sound signals are available as audio files (.wav).

## Supplementary Materials

The following supporting information can be downloaded at: https://www.mdpi.com/article/10.3390/biology13121071/s1, Figure S1: Schematic representation of the recording set-up under laboratory conditions.
