# Correction: Sarria-Sarria et al. A New Genus of Andean Katydid with Unusual Pronotal Structure for Enhancing Resonances. *Biology* 2024, *13*, 1071

**DOI:** 10.3390/biology14030262

**Published:** 2025-03-05

**Authors:** Fabio A. Sarria-Sarria, Glenn K. Morris, Fernando Montealegre-Z

**Affiliations:** 1 School of Environmental and Life Sciences, University of Lincoln, Lincoln LN6 7DL, Lincolnshire, UK; 2 Department of Biology, University of Toronto at Mississauga, Mississauga, ON L5L 1C6, Canada

## Missing Information

In the original publication [1], the type species for *Tectucantus* gen. nov. was not assigned in the Results in Section 3.1.1, before paragraph 1. The assigned type species is *T. tinnulus* and should read as follows:

Type species: *T. tinnulus* sp. nov. here described.

## Text Correction

There was an error in the original publication [1]. There is a Latin grammatical error in the species epithet for *T. planatum*.

A correction has been made to Sections 3.1.4, 3.2 and 3.2.3 of the Results, Section 4.1 of the Discussion; the accurate form should be *T. planatus*. This amendment should be consistently reflected throughout the text, as well as in the labeling and legends of the figures and tables included (Figures 1–7 and Tables 1 and 2).

## Error in Figure/Table

In the original publication [1], there was a mistake in the legends for Figures 1–7 and Tables 1 and 2 as published. There is a Latin grammatical error in the species epithet for *T. planatum*, and the correct spelling should be *T. planatus*. The corrected legends, Figure 1, Figure 2, Figure 3, Figure 4, Figure 5, Figure 6 and Figure 7, and Table 1 and Table 2 appear below.

The authors state that the scientific conclusions are unaffected. This correction was approved by the Academic Editor. The original publication has also been updated.

## Figures and Tables

**Figure 1:**
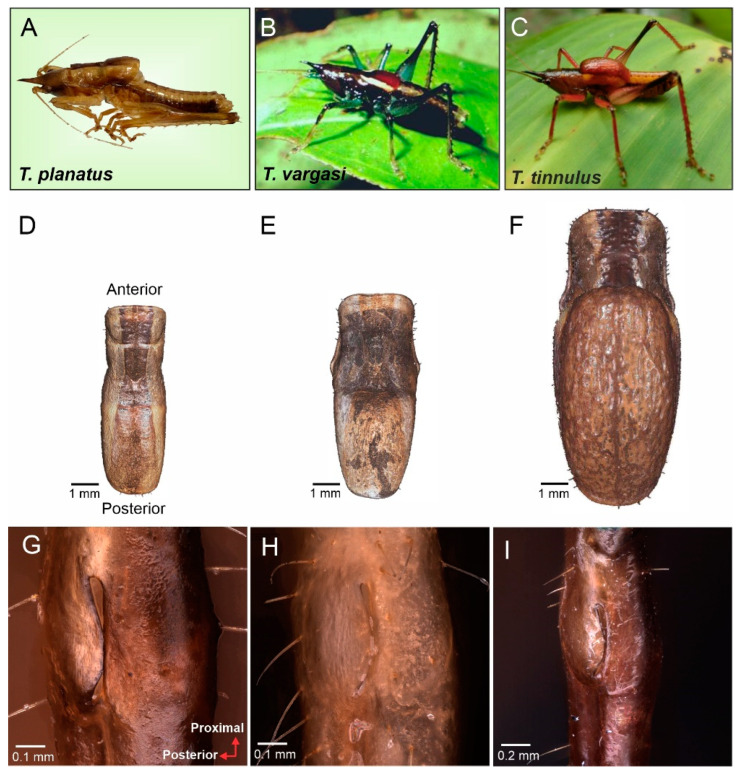
Morphological characters of *Tectucantus* spp. (**A**–**C**) Habitus of male of *T. planatus*, *T. vargasi*, and *T. tinnulus*. (**D**–**F**) Dorsal view and size comparison of pronotum. (**G**–**I**) *Tectucantus* spp. tympanal slits design.

**Figure 2:**
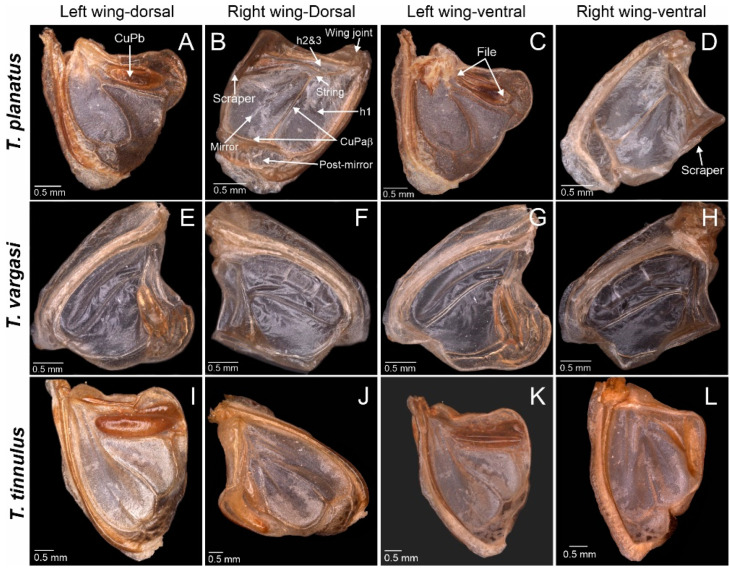
*Tectucantus* spp. wing morphology, dorsal and ventral view. (**A**–**D**) *T. planatus* (**E**–**H**) *T. vargasi* (**I**–**L**) *T. tinnulus*.

**Figure 3:**
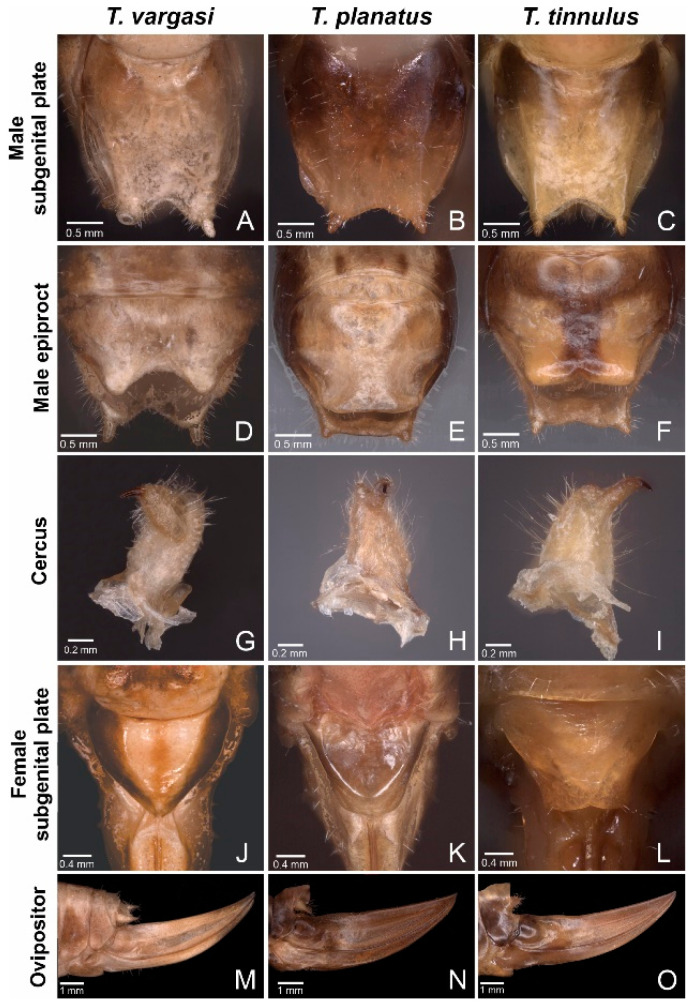
*Tectucantus* spp. abdominal morphological features. (**A**–**C**) Ventral view of male subgenital plate. (**D**–**F**) Dorsal view of male epiproct. (**G**–**I**) Male right cercus. (**J**–**L**) Dorsal view of female subgenital plate. (**M**–**O**) Side view of female ovipositor.

**Figure 4:**
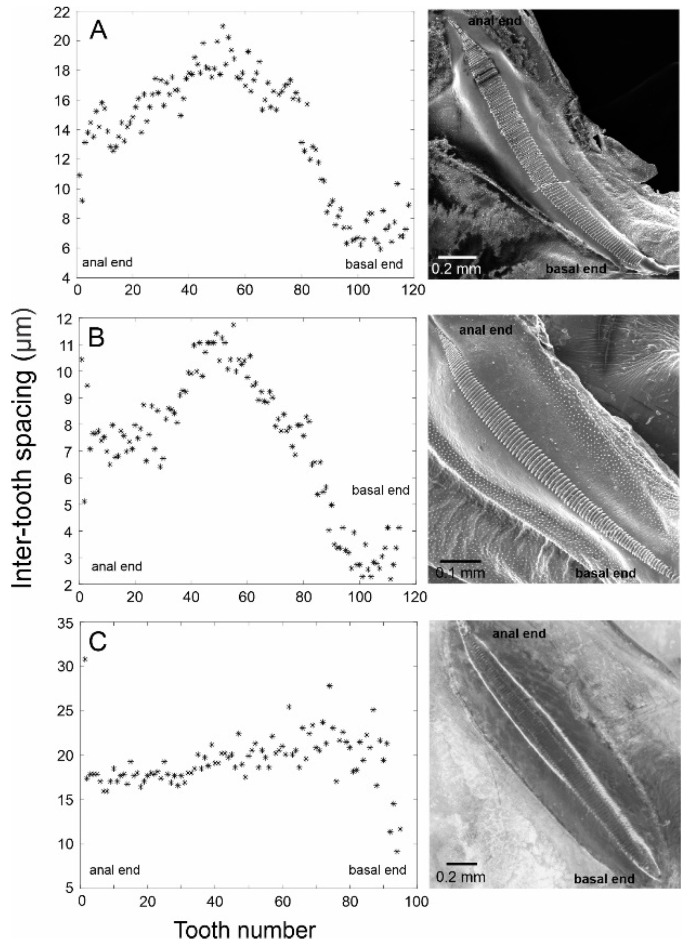
The stridulatory file of *Tectucantus* spp. Graph panels on the left show the measurements of inter-tooth distances in the direction of scraper motion during stridulation (anal to basal), and the panels on the right show SEM pictures of the files of each species, except for *T. tinnulus*. (**A**) *T. planatus*, (**B**) *T. vargasi*, and (**C**) *T. tinnulus*.

**Figure 5:**
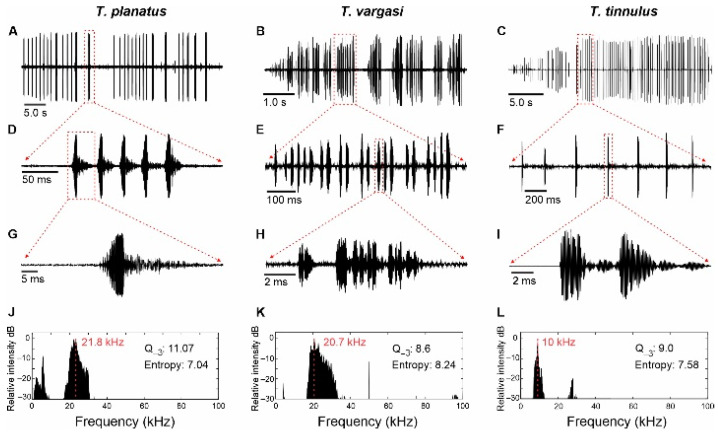
Acoustic analysis of *Tectucantus* spp. (**A**–**C**) Calling song of a recorded section. (**D**–**F**) Close-up view of an echeme. (**G**–**I**) Close-up view of a syllable (**J**–**L**) Frequency spectrum of a single syllable, red dashed line indicates peak frequency.

**Figure 6:**
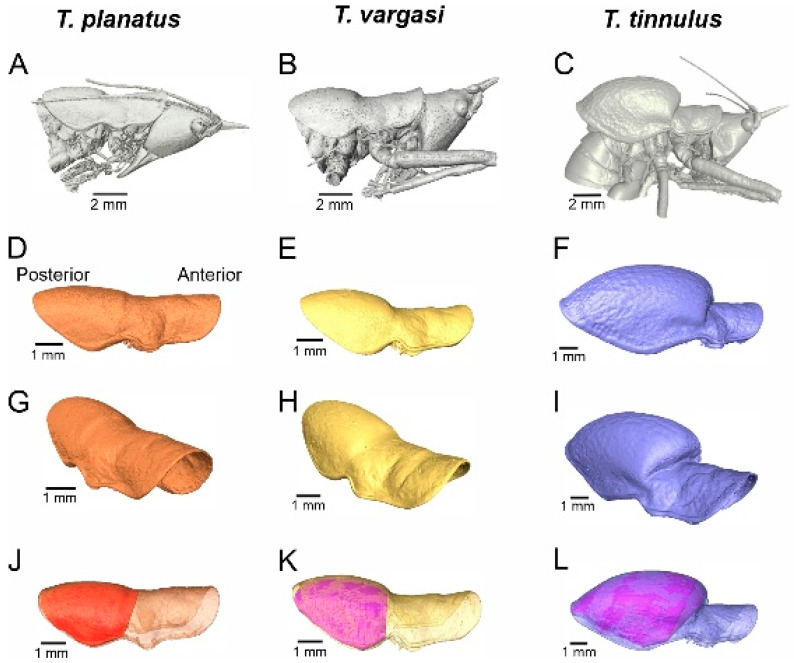
Three-dimensional segmentation and volumetric features. (**A**–**C**) Side view of head and thorax. (**D**–**L**) Pronotum 3D reconstruction, side view and pronotal cavity volume. (**J**–**L**) Pronotal volume represented by the shaded area.

**Figure 7:**
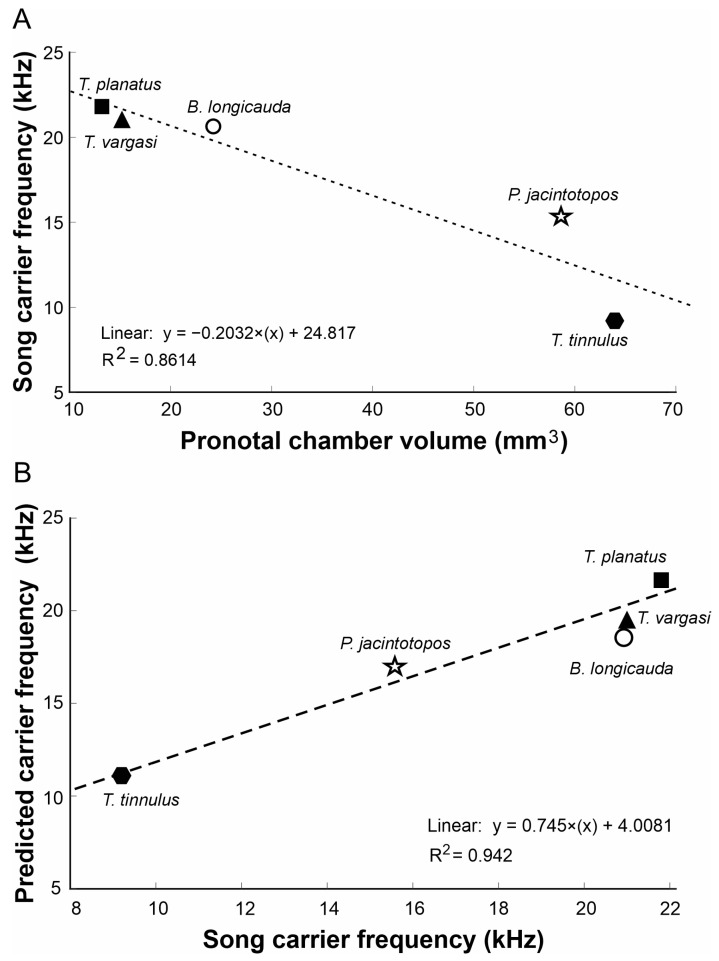
Relationships between *Tectucantus* spp. pronotal cavity and calling songs. (**A**) Correlation between pronotal volume and carrier frequency. (**B**) Correlation between carrier frequency and predicted carrier frequency.

**Table 1:** Body measurements. All measurements are in mm. For paired features (e.g., legs), left/right features were reported, respectively. n.a. = not applicable, where the structure was not present to be measured (e.g., damaged, absent, or absent due to sex differences).

Species	*T. planatus*				*T. vargasi*				*T. tinnulus*			
Body Part	♂ (*n* = 5)	SD	♀ (*n* = 2)	SD	♂ (*n* = 3)	SD	♀ (*n* = 1)	SD	♂ (*n* = 6)	SD	♀ (*n* = 1)	SD
Fastigium	2.18	0.3	2.4	0	2.23	0.28	2.5	0	2.92	0.13	2.63	0
Fastigium base	0.82	0.07	0.81	0.08	0.7	0.07	0.88	0	0.92	0.05	0.95	0
Frons	3.41	0.05	3.88	0.18	3.25	0.28	3.7	0	3.89	0.12	4.3	0
F-Femur	4.98	0.11	5.56	0.05	4.82	0.02	5.25	0	5.65	0.07	5.8	0.07
F-Tibia	5.16	0.11	5.78	0.11	5.07	0.25	5.3	0.07	6.11	0.06	6	0.35
M-Femur	4.95	0.14	5.44	0.48	4.69	0.16	4.95	0.07	5.31	0.11	5.45	0.07
M-Tibia	5.26	0.01	5.6	0.11	4.9	0	5.38	0.18	6	0.11	6.25	0
H-Femur	9.03	1.51	10.6	0.28	9.07	0.17	10.15	0.21	10.03	0.11	10.55	0.07
H-Tibia	9.16	1.39	11.2	0.07	9.18	0.11	10.1	0.14	10.18	0.06	11.15	0.21
Eye	0.9	0.02	0.94	0.01	0.8	0.04	0.9	0	0.9	0.01	0.99	0.02
inter eye space	2.03	0.07	2.25	0	2.08	0.16	2.1	0	2.32	0.1	2.38	0
Pronotum length	6.56	0.2	4.68	0.25	6.55	0.18	4.5	0	9.23	0.4	4.3	0
Pronotum width	3.08	0.14	3.63	0.53	3.13	0.38	3.25	0	3.63	0.13	3.95	0
Subgenital plate length	2.16	0.28	1.2	0	2.16	0.4	1.25	0	2.24	0.1	1.5	0
Subgenital plate width	1.96	0.16	1.5	0.35	1.87	0.4	1.62	0	2.24	0.07	2	0
Epiproct	1.47	0.07	0	0	1.08	0.19	0.62	0	1.24	0.06	0.5	0
Abdomen length	9.28	1.23	12.25	1.48	7.83	1.53	7.5	0	8.57	1.13	11	0
Ovipositor	n.a.	n.a.	7.1	0.57	n.a.	n.a.	6	0	n.a.	n.a.	8	0
Ovipositor base	n.a.	n.a.	2	0	n.a.	n.a.	1.75	0	n.a.	n.a.	2.25	0

**Table 2:** Estimated carrier frequency using Equation (2) for a neckless Helmholtz resonator.

Species	Volume (mm^3^)	Radius (mm)	CF Song (kHz)	Predicted Resonant Frequency (kHz)
*T. tinnulus*	63.95	1.39	9.5	10.4
*T. vargasi*	15.22	1.05	21	19.5
*T. planatus*	13.2	1.06	21.8	21
*B. longicauda*	24.48	1.47	20.8	18.2
*P. jacintotopos*	58.71	3.02	15.6	16.8
