# Supplementary figures and images for: A New Genus of Andean Katydid with Unusual Pronotal Structure for Enhancing Resonances
Source: Biology (Basel). 2024 Dec 20;13(12):1071. doi: 10.3390/biology13121071 (PMC11672931; doi:10.3390/biology13121071)

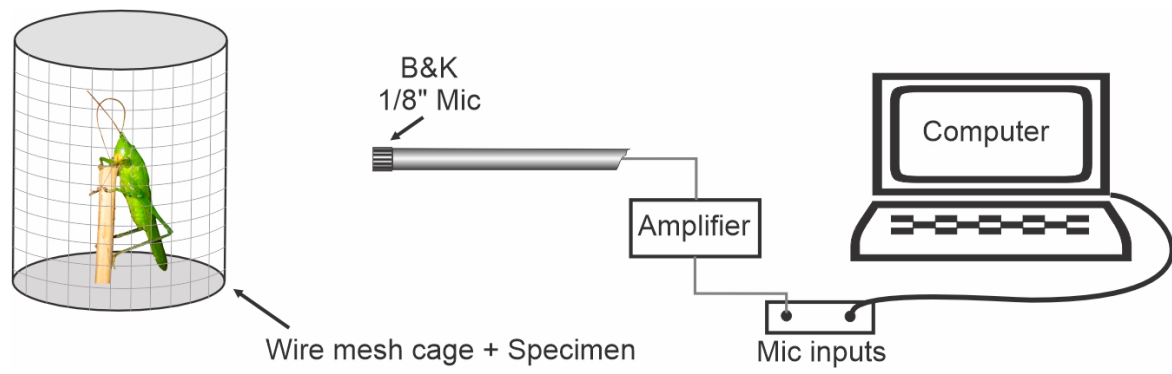

**Figure S1.** Schematic representation of the recording set-up under laboratory conditions.

Supplement: Supplementary file 1 [file biology-13-01071-s001.zip › biology-3355446-supplementary.pdf]
